# Discharge criteria from nephrology follow-up back to primary care in modern-era CKD: a review of main guidelines

**DOI:** 10.1093/ckj/sfag057

**Published:** 2026-02-20

**Authors:** Nestor Oliva-Damaso, Lucia Martinez-Palma, Elena Oliva-Damaso, Juan Payan, Richard J Glassock

**Affiliations:** Division of Nephrology, Department of Medicine, Hospital Universitario Costa del Sol. Marbella, Málaga, Spain; Department of Medicine, Primary Care, Las Lagunas, Fuengirola, Malaga, Spain; Division of Nephrology, Department of Medicine, Hospital Doctor Negrin, Las Palmas de Gran Canaria, Spain; Division of Nephrology, Department of Medicine, Hospital Universitario Costa del Sol. Marbella, Málaga, Spain; Department of Medicine, Geffen School of Medicine at UCLA, Los Angeles, CA, USA

To the Editor,

Chronic kidney disease (CKD) affects >700 million, people globally and represents a public health and economic challenge. CKD contributes significantly to morbidity, premature mortality and increasing healthcare costs, while its impact is amplified by population aging, demographic growth and disparities in healthcare infrastructure [1, 2]. In response, several clinical practice guidelines (CPGs) have been developed to standardize evidence-based strategies for CKD and to optimize kidney treatment. These therapeutic advances suggest that partial regression or stabilization of renal function, even in advanced CKD stages, is increasingly achievable in selected patient populations [3]. While referral criteria to nephrologists are well established [4], standardized discharge back to primary care or shared-care criteria are rarely addressed in CPGs. As earlier detection and improved therapies expand the number of stable or regressing patients, the absence of standardized discharge or shared-care criteria creates variability in clinical practice and inefficiencies in healthcare resource utilization, an organizational problem that if sustained can become a pivotal issue in countries with ‘overloaded’ public healthcare systems.

To explore this issue, we conducted a structured review of nephrology clinical practice guidelines published between 2019 and 2025, searching major guideline repositories and databases. Eligible documents addressing adult CKD were based on systematic evidence synthesis with graded recommendations, available in English or Spanish. Paediatric, transplant, dialysis-focused, consensus-only and non-guideline documents were excluded. Data were extracted regarding referral and discharge criteria, shared-care recommendations and communication strategies between nephrology and primary care. Seven major clinical practice guidelines met the inclusion criteria [Kidney Disease: Improving Global Outcomes (KDIGO) 2024, Kidney Disease Outcomes Quality Initiative (KDOQI), National Institute for Health and Care Excellence NG203 (2021), CARI Guidelines, the Sociedad Española de Nefrología (SEN, 2023), the UK Kidney Association and the Swedish Renal Registry recommendations (2022)].

Across these guidelines, all emphasize early detection, risk stratification and individualized management, but the degree of explicit discharge guidance varies substantially (Table 1). The KDIGO 2024 guidelines promote collaborative, risk-based management and highlight tools such as the Kidney Failure Risk Equation (KFRE) to guide follow-up intensity, yet it does not define formal discharge thresholds. Similarly, the KDOQI stresses integrated care and shared responsibility without specifying reverse-referral criteria. In contrast, the National Institute for Health and Care Excellence (NICE) provides measurable parameters for reallocation to primary care, recommending that patients with stable estimated glomerular filtration rate (eGFR) >45 ml/min/1.73 m^2^ for at least 12 months, limited albuminuria and no progression may be safely managed outside nephrology, with clear re-referral triggers. The CARI guidelines adopt a stepped shared-care model in which management intensity aligns with disease severity and stability, embedding discharge within a continuum rather than treating it as a discrete event. The SEN consensus explicitly endorses primary care follow-up for non-progressive CKD G1–G3a and for selected elderly or frail patients with stable advanced disease and no indication for renal replacement therapy. The UK Kidney Association integrates risk stratification, including low predicted kidney failure risk, with structured discharge pathways and defined communication standards. The Swedish recommendations provide explicit criteria—preserved or stable kidney function, minimal albuminuria, absence of metabolic complications and sustained stability for at least 12 months—and uniquely incorporate teleconsultation pathways to ensure continued access after discharge.

**Table 1:** Summary of CKD guidelines including discharge or shared-care criteria between nephrology and primary care.

Guideline	Year	Scope/region	Discharge or shared-care criteria	Risk tools or follow-up framework	Telemedicine/re-referral protocols
KDIGO	2024	International	No explicit discharge section. Collaborative management recommended for CKD G1–G3a, albuminuria <30 mg/g, stable comorbidities and no progression	Risk-based follow-up using KFRE; integration of multidisciplinary care	Not specified. Encourages communication and local adaptation
KDOQI	2020	USA	No formal discharge criteria. Early CKD (stages 1–2) can be managed in primary care	Shared, integrated care based on disease stability and patient engagement	Not specified. Emphasis on coordinated care networks
NICE NG203	2021	UK	Explicit: Discharge possible when eGFR >45 ml/min/1.73 m^2^, stable ≥12 months, albuminuria <70 mg/mmol and no progression	Annual primary care review; re-referral if function decreases or albuminuria increases	Embedded within NHS referral protocols
CARI	2022	Australia/New Zealand	Stepwise model: Mild/stable CKD (eGFR >45 ml/min/1.73 m^2^, ACR <30) in PC; moderate cases shared; advanced remain in nephrology. Discharge possible after stabilization and education	Shared-care ladder; emphasizes patient education and local monitoring capacity	Not specified, but encourages communication pathways
SEN	2023	Spain	Explicit: Follow-up in PC for CKD G1–G3a without progression, elderly (>80 years) with stable GFR <30 ml/min/1.73 m^2^ or frail patients under remote supervision	Shared-care approach incorporating age, frailty and stability	Includes remote monitoring and digital communication tools
UKKA	2020–2023	UK	Explicit: Follow-up in PC for low–moderate risk (KFRE <3%) and stable cardiovascular/metabolic control	Structured discharge pathways; multidisciplinary communication	Defined re-referral criteria and digital care coordination
Swedish Renal Registry	2022	Sweden	Explicit: GFR ≥45 ml/min/1.73 m^2^, ACR <30 mg/g, no anaemia or CKD-MBD and no progression ≥12 months	Registry-based follow-up with standardized risk thresholds	Includes teleconsultation protocols for discharged patients

ACR: albumin:creatinine ratio; CKD-MBD: chronic kidney disease–mineral and bone disorder; PC: UKKA: UK Kidney Association.

Although substantial advances in pharmacotherapy have improved renal and cardiovascular outcomes, the organization of care remains uneven, particularly regarding transitions between nephrology and primary care. A new paradigm changing classical progression of CKD to ‘remission’ is emerging [5] where the use of optimized nephroprotective medications (ACEi/ARB+SGLT2i+-finerenone+-GLP1a) enables a new scenario in the clinical kidney office as discharge back to primary care. In countries with a public healthcare system, discharging CKD patients—with no progression (low KFRE), optimized kidney treatment and minimum albuminuria—may leave space for new non-evaluated patients. An international trend toward structured reverse referral (discharge to primary care) and continued shared CKD care models are emerging, emphasizing stability, age, risk of progression and patient education. Nevertheless, uniform discharge frameworks are generally lacking. Future CPGs should integrate standardized criteria and interpractice-level communication pathways to enhance continuity and optimize nephrology resources.
